# Mr. Salmon on Stricture of the Rectum

**Published:** 1828-04-01

**Authors:** 


*A Practical Essay on Stricture of the Rectum; illustrated by Cases, shewing the Connexion of that Disease with Affections of the Urinary Organs and the Uterus—with Piles, and various Constitutional Complaints.* By Frederick Salmon, Surgeon to the General Dispensary, Aldersgate Street, and formerly House-Surgeon to St. Bartholomew's Hospital. Octavo, pp. 188. London, 1828.

It is no doubt true, that strictures in the urethra are infinitely more frequent than strictures in the rectum. But when these last do occur, (which, by the bye, is by no means a rare case,) they are a great deal more distressing to the patient, and detrimental to the constitution, than the former. The stomach and bowels are organs of higher importance in the animal economy than the kidneys; and, consequently, whatever disturbs their complicated functions, whether in the process of digestion, or in the evacuation of the fæcal remains, produces more serious and more numerous sympathetic disorders in the constitution at large, than any mechanical difficulty which the bladder may experience in the evacuation of the renal secretion. We are not unaware, indeed, that the irritation of an urethral stricture will occasion, in some people, considerable disturbance of constitution; but we do maintain, that these sympathetic disturbances are more distressing than dangerous—and that, in the worst cases, they are not to be compared with the effects of stricture in the rectum.

The object of Mr. Salmon is to prove, that stricture of the rectum is a very common disease, inducing other important affections—and that surgery furnishes us with means adequate to its removal or alleviation, if judiciously exercised. It is not our intention to enter into a minute analysis of Mr. Salmon's book in this place; but merely to touch lightly on some of the principal features of the work.

Under the head of "Causes and Kinds" of stricture in recto, Mr. S. examines the opinions of different surgeons. Mr. Bell considers the stricture as owing to a morbid change in the inner membrane of the intestine—not unfrequently about the inner edge of the sphincter ani. Mr. Copeland thinks the complaint may be produced by whatever excites inflammation or irritation in the inner membrane of the canal. Mr. White (of Bath) thinks the predisposing cause is the narrowing of the gut about the termination of the sigmoid flexure of the colon—which narrowing he seems to look upon as sometimes an original malformation. Mr. Salmon is disposed to agree, in some measure, with Mr. White, on this subject, and thinks it illustrates a fact he has repeatedly noticed—that of several children in the same family being afflicted with stricture.

"Any cause, however, tending to produce local irritation in the rectum, existing for a continued period, may give rise to contraction: thus, habitual costiveness is among the most frequent causes of the complaint.

"Another, and I believe a common, cause of stricture, will be found in the administration of large doses of drastic purgative medicines, a practice peculiarly tending to irritate the bowels, and, by promoting increased contractile action, to impair their natural functions. Patients have informed me, that the first symptoms of the disease followed immediately upon taking violent aperients.

"Indigestion may give rise to stricture, the acrid state of undigested matter irritating the inner coat, and, in this manner, causing improper action of the intestine."

A peculiar constriction of the anus is sometimes the cause of stricture. Mr. S. has rarely found this constriction unattended with obstruction very high up in the rectum. In females, an occasional and afflicting source of stricture is an enlarged and tender condition of the uterus, of which we have a melancholy case now under our care. Every time that a motion is procured, by nature or art, the sufferings are dreadful, and can only be allayed by the introduction of laudanum and starch into the rectum. In these unhappy cases, little or nothing can be done by surgery. The only means of mitigating the pains, are, by emollient injections, to bring away the fæces in a soft state, and afterwards throwing up anodynes.

Strictures may occur from enlargement of the prostate gland—from piles, tumours, or excrescences—in short, from any cause, constitutional or mechanical, creating irritation in the rectum, and, thus, ultimately inducing spasmodic action of its muscular coat. In process of time, the complaint changes from spasmodic constriction into permanent stricture. Our author, indeed, thinks that the greater number of strictures are, at first, simply spasmodic, but that, in time, depositions take place between the coats of the bowel, that assist in preventing the natural action of the part, ending ultimately in induration and thickening, so that all trace of the natural structure of the gut is lost.

The third chapter of the work is on the SYMPTOMS of Stricture. Insidious as are the signs of this disease, in its early stage, yet Mr. Salmon thinks they may be detected by close examination. We give the following extract from his symptomatology; observing, however, that many of the symptoms here laid down do frequently occur, independently of any stricture in the rectum.

"In its commencement, trifling irregularity of the bowels occurs, the motions being deficient in quantity, sometimes passed in small pellets, at others flattened like tape, or having the appearance of worms. Instead of the bowels being every day fully and freely emptied of their contents, a day, sometimes two, will now and then intervene without any evacuation occurring; or, what is more common, patients following their customary habits, will, at a particular period of the day, void, as they suppose an adequate motion from their bowels; yet, were they to examine the quantity, they would find it next to nothing. A sense of soreness is experienced at the anus, at the verge of which, after the discharge of the evacuations, the skin, from extreme rigidity of the part, frequently gives way, forming several small cracks or fissures distressingly annoying. Occasional pains occur in the loins and lower part of the back, which sometimes extend into the groins, particularly the left, the hips and thighs, imparting a sensation similar to cramp.

"Sooner or later decided costiveness supervenes; opening medicines are resorted to, which, affording temporary relief, satisfy the patient and his medical friend. This state of things will sometimes exist for a very extended period—for months, nay, even for years, people will from day to day administer opening medicines, increasing the quantity in proportion as their constant use and the progressive advancement of the disorder render them necessary, till at last, weary of the trouble and inconvenience, they relinquish them. Very speedily this irregularity is followed by a difficulty of passing the contents of the bowels; after going to stool, a sensation is experienced as if the rectum was not completely emptied; persons feel a disposition, yet have not the capability to pass more relief, and endeavour by violent straining to force out the contents of the gut, which proves of little avail, serving only to produce a discharge of blood, and a prolapsed state of the bowel. As the disorder advances, these symptoms progressively increase, till at last, many days together will pass without any relief occurring, though the patient will be tormented by frequent calls, and the most painful yet ineffectual efforts."

In other instances (of which we shall presently give a remarkable example) accumulations take place in the colon, distending it to an enormous size—the whole alimentary canal becomes disordered—and inflammation of the bowels ensues, too often terminating in death. The various sympathetic disorders that are consequent on stricture in the rectum, cannot here be portrayed. The stomach, the kidneys, the bladder—in short, the general health, all suffer from this terrible disease.

TREATMENT. Mr. Salmon divides this into constitutional and local. In the former, however, he includes leeching the anus, cupping the perineum, &c. The main object is attention to the bowels.

"No use of instruments can possibly establish a recovery, unless the bowels are brought to a free and comfortable relief daily; to accomplish this, two points should constantly be observed—first, that we do not load and annoy the stomach by the too plentiful administration of food—secondly, that we do not irritate the bowels by an injudicious use of purgative medicines. By adopting the plan of diet hereinafter advised, we shall avoid the first evil, but the administration of medicine is of equal importance. Here I cannot too strongly deprecate the common every day's practice of giving violent doses of purgative medicine; not only is no benefit derived from such treatment, but serious injury is frequently induced. When adopted in diseases of the rectum such must be the result, since large quantities of feculent matter are driven from the small intestines into the colon, already distended, as a consequence of stricture in the rectum, and highly irritated, and thus arise frequent and distressingly painful efforts to pass motions, the straining to accomplish which, may, in extreme instances, induce inflammation, or even rupture of the intestine at the sigmoid flexure."

Here Mr. Salmon introduces the case of a lady, who died from the effects of violent purgative medicines. In a severe attack of constipation, peritonitis supervened, for which drastic purgatives were exhibited. In straining at stool, she felt something give way internally, and quickly expired. On dissection, it was found that a cherry-stone had lodged in the sigmoid flexure of the colon, where the passage was nearly obliterated by a stricture, close to which the gut had given way, and extravasation was the result. Castor oil, or the electuary of senna with sulphur, are no doubt, the best aperients, in cases of stricture. The use of injections are here of great importance; but the way in which they are employed often frustrates the intention of the practitioner. Half or three parts of a pint of thin gruel with a little castor or common oil, should be thrown gently up, and the patient should keep in bed for an hour or two afterwards, so that it may be retained in the bowels, and thus soften the fæces before they pass the stricture. It is certainly a matter of regret, as our author observes, that the fastidious feelings of some, and the want of consistent recommendation in others, should prevent the adoption of this salutary and harmless measure. We copy our neighbours in many of their frivolities and inconsistencies, yet disdain to adopt those habits and customs that are both healthy and cleanly.

On the subject of diet, Mr. Salmon makes many judicious observations, and shews himself a warm advocate of the Abernethian doctrines.

THE BOUGIE. Mr. Salmon thinks that the constitutional treatment, however careful, can be only palliative, without the bougie. The following directions are judicious, and they are too little attended to by surgeons in general, who introduce the bougie with little or no preparation.

"One or two hours previous to the examination of the rectum, an injection is to be administered of tepid poppy water, containing forty or fifty drops of laudanum, which will tranquillize the bowel, and remove any lodgement of fæces. The patient should also be requested to make water immediately previous to the introduction of the instrument. The rectum is first to be examined with the finger, to ascertain that there is no kind of obstruction near the orifice.

"The patient, if a male, leaning over the back of a chair, or the side of a bed,* should draw aside the nates fairly to expose the orifice of the bowel. A full sized bougie, not less than eleven inches in length, thoroughly softened, and well oiled, adapted to the shape of the passage through which it is to be passed, is to be introduced, with the convexity of the first curve towards the sacrum, in which way it is to be passed upwards and backwards about two inches,† through the third portion of the bowel, provided it gives no pain, for the introduction will commonly produce an uneasy sensation; we continue to propel the bougie in the same direction, about three or three and a half inches higher, or through the second portion of the rectum; the point of the instrument will now bear directly upon the hollow of the sacrum, and the but-end towards the left side of the body. With a view, therefore, of avoiding the sacrum, and of accommodating the instrument to the great curve of the rectum, we change its position, by describing the segment of a circle from left to right, with the but-end, turning it upwards, at the same time continuing to propel the instrument. Having described this segment, we shall have carried the bougie full four inches farther, or to what may be considered the extent of the rectum. But it is yet to be introduced into the sigmoid flexure; we therefore triflingly depress the but of the instrument, at the same time propelling it upwards, till the whole is fairly within the sphincter—this accomplished, we may be satisfied."

The patient generally complains of pain, both in the rectum and over the surface of the abdomen, when the instrument has passed about five or six inches up. If obstruction be encountered, trifling pressure is to be maintained, for a minute or two, and then, if the pain increase, and the instrument remain

"* In females the examination being made beneath the bed clothes, is conducted without the slightest exposure."

"† The last curve of the rectum is so trifling, that it matters not whether we introduce the convex or concave part of the instrument towards the sacrum, but by passing it with the convexity backwards, we avoid the necessity of altering the position of the bougie in passing it through the second curve of the bowel."

stationary, it is to be withdrawn—a smaller size introduced—and so on, from larger to smaller, till we ascertain the bougie that passes with trifling pain or difficulty into the sigmoid flexure. The instrument should remain in the bowel ten or fifteen minutes, provided it produces no considerable irritation; at the expiration of which time it is to be removed, and allowed to harden in the shape which it had assumed in the intestine. The first introductions generally produce some irritation, and a nisus to relieve the bowels; but the rectum gets accustomed to the bougie in a short time. The operation should be repeated at intervals of three, four, or five days, gradually increasing the size of the bougie, and lengthening the period of its remaining in the bowel.

In the foregoing directions, it will be seen that Mr. Salmon differs from most preceding writers, as to the frequency of repeating the operation. Mr. Bell, after stating the constitutional treatment, directs a daily introduction of the bougie. Mr. Copeland gives similar instructions, but recommends the bougie to be left in for a longer period. Mr. White says the instrument should not remain longer in than half an hour at first—but, after some introductions, he lets it stay in the rectum eight or ten hours at a time. In general he uses the bougie daily. Against this diurnal introduction, the following objections are urged.

"The action resulting from the introduction of the bougie is twofold—dilatation and absorption. Now it must be obvious, that to produce these effects, it is necessary that a certain degree of pressure be maintained upon the strictured portion of the gut. It is by dilatation that the simple spasmodic stricture is removed, and by dilatation and absorption combined, that the permanent obstruction will be materially alleviated, if not totally cured. Our object should certainly be to dilate the passage as speedily as possible, nevertheless it should be borne in mind, that the introduction of the instrument causes a specific action or irritation, by which we overcome the unhealthy function of the part; this action, or irritation, should be allowed totally to subside before we again introduce the bougie. If, however, we daily pass bougies, suffering them to remain in the bowel for eight and ten hours at a time, I would ask, what time do we allow for the subsidence of the irritation we have created? nay, do we not encounter it at every use of the instrument, and thus rather promote than lessen the disease?

In the early period of his practice, Mr. S. followed the directions of the writers above-mentioned—experience induced him to prefer the plan which we have noticed.

The structure of the bougie is considered to be a matter of great importance. He thinks Mr. White's bougie is not sufficiently firm. It yields too readily, and consequently does not make pressure enough on the strictured parts.

"The instrument I have been accustomed to use, is composed of fine linen cloth, very heavily coated with wax, and a certain portion of diachylon plaster, mixed with a small quantity of lamp black. From immersion in very hot water, some minutes previously to being used, it is rendered soft and pliable to any extent, nevertheless retaining one regular and smooth surface. When it is introduced into the bowel, instead of becoming softer, it hardens to a degree sufficient to afford considerable resistance to the action of the stricture."

As to gum-elastic bougies, when the stricture is remote from the orifice, it is next to impossible to introduce them. Metallic ones are still worse.

The introduction of the bougie is followed by cramps in the thighs and legs —numbness—shiverings—sickness, &c. not very unlike those phenomena which follow the introduction of a bougie into the urethra. These disappear after a few operations with the instrument.

"Of all the annoyances attendant upon the introduction of the instrument, none is more troublesome to the patient and the surgeon than the powerful action and the irritation of the sphincter muscle; which may be considerably lessened by taking care to pass the bougie completely through both the external and internal sphincters, first affixing a tape through its loop, a precaution which should never be neglected; for the bowel will sometimes draw up the bougie, so as to take it completely out of the reach of the finger; this happened in Case the Seventh, hereinafter narrated. The patient, exceedingly alarmed, sent for me; but, prior to my arrival, he had voided the bougie, rolled up like a ball. It caused the most distressing pain in its passage through the sphincter, but no subsequent serious inconvenience resulted from the accident."

It is curious that patients under the use of the bougie are more susceptible of cold than at other times. In some cases, the bowels will soon become regular after the use of the bougie—in others, no material benefit will result for many weeks—in some, the bowels never become regular, however much the health may be improved.

In a very short chapter on carcinomatous disease of the rectum, Mr. S. advises, of course, that we should avoid producing irritation by any attempt at introducing bougies. All we can do, in these unhappy cases, is to sooth pain, and keep the bowels gently open.

A considerable number of valuable cases are introduced into the work by Mr. Salmon; but we shall only have room to notice one, which is very curious and interesting. We must abridge it very much.

*Case.* Mr. ———, aged 41, complained, on the 31st of October, 1821, of pains in various parts of the body, especially the loins, knees, and ankles. He had an ulcerated throat, and his digestion was greatly deranged. The least quantity of food produced pain in his stomach. He had undergone courses of mercury, under the idea that he had syphilis, and he was now taking four grains of calomel daily. The mercury was discontinued—proper medicines were prescribed—and, on the 24th Dec. we find him reporting himself quite well, and setting out on a journey. On the 13th May, 1822, Mr. Salmon saw him, when he informed Mr. S. that, till within a fortnight, he had been in perfect health. He now laboured under constipation and irregularity of the bowels—hæmorrhage occasionally from the rectum—pain in the region of the stomach—scanty lateritious urine—night-sweats—cough. The pulse was tranquil. He had been a free liver, and even now indulged in wine. He got a little better, and went to Brighton, on returning from which place, in July, 1822, he presented a very altered appearance. He was emaciated in the face and limbs, while the abdomen was enlarged and tender, but without distinct fluctuation. He suffered much when his bowels were moved. It was apprehended that his liver was diseased, and mercury, squills, &c. were prescribed. On the 27th July, fluctuation was evident, and he was generally anasarcous. He was seized with violent pain in the abdomen, severe purging, and profuse hæmorrhage from the bowels. All things went on badly, and, on the 2d September, it was determined to tap the abdomen. On examination, a hard protuberance was felt under the margin of the ribs, on the left side. It was considered by Mr. S. and a physician to be an enlarged liver. On the 10th September he was supposed to be dying. He was insensible—pulse intermitting. Brandy recruited him, and, on the 11th, he was much better, having had a diarrhœa, accompanied with much hæmorrhage, and immense *prolapsus ani.* On the 1st October, Mr. S. examined the rectum, and a firm stricture was discovered a few inches up. Some oil and water gruel were injected, when a large quantity of horribly offensive fæces came away. The next day a still larger collection was discharged, the fæces resembling sheep's dung. In short, there had been a complete Augean stable here, in the form of an immensely distended colon. On the 22d Oct. we find the patient greatly improved, by the use of injections and bougies. By the 15th November, he was able to go out, and take exercise—his belly was reduced—his bowels acted regularly, but the motions were still followed by blood. On the 13th December, we find him residing in a tavern in London, indulging in every kind of excess. The swelling of the legs had returned, and the distension of the colon was evident—the bowels were very irregular and the evacuations trifling. We must pass over a great variety of vicissitudes which this miserable and imprudent patient experienced, between December, 1822, and June, 1823, when he suddenly expired, while on the close-stool. The following examination will elucidate this strange eventful history.

"Upon opening the abdomen, I discovered one of the most extraordinary specimens of disease I have ever witnessed; no vestige of the stomach, liver, or small intestines could be seen, but one immense tumour, having an irregular surface, in some points perfectly hard, in others of a softer consistence. Upon examination, this proved to be the colon, distended with hardened fæces; so immensely large was it, that the transverse arch of the bowel extended within a trifling distance of the *pubis*, and the ascending and descending portions nearly coalesced; from these several points coagulated lymph had been thrown out, which had become organized, connecting the different portions of the gut, which was, as it were, glued in one mass: at the superior part, the colon firmly adhered to the great arch of the stomach, thrusting this organ, together with the liver, under the ribs; it had also contracted firm adhesions to the liver and diaphragm; at the sigmoid flexure it was united in the same manner to the superior aperture of the *pelvis*, and its lateral portions to the abdominal *parietes*. After a laborious dissection, I succeeded in detaching it from its different adhesions, and emptied it of the fæces with which it was distended; this I could only do by cutting it through, above the sigmoid flexure and *caput coli*; at the former of which points, the bowel was so completely obstructed, that even water would not pass through it. The intestine, notwithstanding its distension, was very considerably thickened through its whole extent. This appeared principally to have resulted from deposition between the muscular and mucous coats. In the rectum there were two obstructions, one about four inches from the *anus*, and a second at about seven inches; and at the sigmoid flexure I could scarcely find any passage at all. The appearance of the obstruction at the sigmoid flexure was not the same as that in the rectum; the former being perfectly circular, whereas the latter was more like the elongation of the semilunar folds of the bowel, the general structure of which was materially thickened through the whole of the gut; the colon was likewise violently inflamed throughout the inner surface, there being here and there patches of ulceration; the small intestines were glued together in one mass, feeling like hard chords, and were diseased through their whole extent. The liver was harder than usual, yet could not be said to be diseased, though it was of a remarkably small size; the gall-bladder was distended with healthy bile, and the stomach contained a considerable quantity of the same fluid. The spleen, pancreas, kidneys, and bladder, were perfectly sound and healthy, as were the lungs and heart, nor was there any unusual quantity of fluid in the pericardium; the cavity of the abdomen contained about three quarts."

The above very curious and interesting case shews how great a mass of feculent matter will lodge in the bowels, while we are daily administering purgative medicines! It also shows that a distension of the colon may be mistaken for enlargement of the liver, or other organ in the abdomen. From this distension arose the difficulty of breathing, the irritation of the bladder, and the numerous indescribable symptoms under which this wretched patient so long laboured.

We must now close our analysis of Mr. Salmon's little work, which is indicative of sound judgment, liberality of sentiment, and a fair portion of practical observation.

